# Retraction: Improvement of therapeutic effects of mesenchymal stem cells in myocardial infarction through genetic suppression of microRNA-142

**DOI:** 10.18632/oncotarget.28819

**Published:** 2025-12-31

**Authors:** Liu-Xue Yang, Chun-Ling Wei, Man-Li Guo, Yong Zhang, Feng Bai, Shao-Gang Ma

**Affiliations:** ^1^Department of Endocrinology and Metabolism, The Second Hospital Affiliated to Guilin Medical College, Guilin 541100, China; ^2^Department of Endocrinology and Metabolism, Huai’an Hospital Affiliated to Xuzhou Medical College, Huai’an Second People’s Hospital, Huai’an 223002, China; ^3^Department of Endocrinology and Metabolism, Suqian People’s Hospital, Nanjing Drum Tower Hospital, Suqian 223800, China; ^*^These authors contributed equally to this work

**This article has been retracted:** Oncotarget has completed its investigation of this paper. It was determined that multiple instances of image duplication and overlap with unrelated papers were found. Specifically, we identified the following issues:

· Figure 2B: Alcian blue IHC image overlaps with an image in Figure 1B of reference [1].

· Figure 2D: The CXCR7 western blot image was reproduced in the 2019 article Figure 5C, labeled as CXCR4 [2].

· Figure 3B: The flow cytometry images in the 3rd panel (Hypoxia+MSCs) and 5th panel (Hypoxia+MSCs-C7) duplicate earlier published images found in Figure 3A of reference [3].

· Figure 4E: The image of mice myocardial infarction (MI) heart (MI+mdMSCs group) duplicates image in Figure 4B of published earlier paper [4], representing rat infarcted heart from C-BMSCs group.

We have attempted to contact the authors regarding these findings multiple times without response. Finally the Editorial decision to retract this article has been made.

Original article: Oncotarget. 2017; 8:85549–85558. 85549-85558. https://doi.org/10.18632/oncotarget.20935
